# Influence of Six Carbon-Based Nanomaterials on the Rheological Properties of Nanofluids

**DOI:** 10.3390/nano9020146

**Published:** 2019-01-24

**Authors:** Javier P. Vallejo, Gaweł Żyła, José Fernández-Seara, Luis Lugo

**Affiliations:** 1 Departamento de Física Aplicada, Facultade de Ciencias, Universidade de Vigo, E-36310 Vigo, Spain; jvallejo@uvigo.es; 2 Área de Máquinas e Motores Térmicos, Escola de Enxeñería Industrial, Universidade de Vigo, E-36310 Vigo, Spain; jseara@uvigo.es; 3 Department of Physics and Medical Engineering, Rzeszów University of Technology, 35-959 Rzeszów, Poland; gzyla@prz.edu.pl

**Keywords:** nanofluid, rheological behaviour, viscosity, carbon nanomaterials, ethylene glycol, water

## Abstract

Nanofluids, dispersions of nanosized solid particles in liquids, have been conceived as thermally-improved heat transfer fluids from their conception. More recently, they have also been considered as alternative working fluids to improve the performance of direct absorption solar thermal collectors, even at low nanoadditive concentrations. Carbon-based nanomaterials have been breaking ground in both applications as nanoadditives during the last decade due to their high thermal conductivities and the huge transformation of optical properties that their addition involves. In any application field, rheological behavior became a central concern because of its implications in the pumping power consumption. In this work, the rheological behavior of four different loaded dispersions (0.25, 0.50, 1.0, and 2.0 wt%) of six carbon-based nanomaterials (carbon black, two different phase content nanodiamonds, two different purity graphite/diamond mixtures, and sulfonic acid-functionalized graphene nanoplatelets) in ethylene glycol:water mixture 50:50 vol% have been analysed. For this purpose, a rotational rheometer with double cone geometry was employed, which included a special cover to avoid mass losses due to evaporation at elevated temperatures. The flow curves of the twenty-four nanofluids and the base fluid were obtained by varying the shear rate between 1 and 1000 s^−1^ for seven different temperatures in the range from 283.15 to 353.15 K. The shear-thinning behaviors identified, as well as their dependences on carbon-based nanomaterial, concentration, and temperature, were analyzed. In addition, oscillatory tests were performed for samples with the clearest Non-Newtonian response, varying the deformation from 0.1 to 1000% with constant frequency and temperature. The dependence of the behaviors identified on the employed carbon-based nanomaterial was described.

## 1. Introduction

Non-renewable reserves like petroleum, natural gas, and coal are the main energy resources for worldwide economy activity. However, their identification as one of the main causes of climate change, the disappearance of some species of animals and plants, and the risk factors for human health has led to a continuous search for alternative energy resources during the last decades [1,2]. Thus, the economic policies of many countries seek the long-term sustainability of their economies and renewable energies are being incentivized as substitutes for fossil fuels. As stated by the REN21 Renewables 2018 Global Status Report [3], among the total energy consumption in 2016, around 79.5% was produced by fossil fuels and 18.2% by renewable energies. Hydraulic energy, wind turbines, solar power, and geothermal energy represented 51%, 25%, 19%, and 0.6%, respectively, of the total renewable electric power production in 2017 [3].

Among the final uses of total energy consumption, heat transfer applications play a main role. In total, 48% is consumed directly as heat, 32% in transport, and 18% as electrical power [3], with heat transfer processes being involved in the three sectors. Therefore, any performance enhancement of the heat transfer processes implies a great leap in terms of energy efficiency. Many advances have been made regarding investing in the improvement of heat exchangers by increasing the heat transfer surface or designing better distribution elements, such as headers, tanks, or inlet and outlet nozzles [4]. Nevertheless, the weak point of these processes remains in the feeble thermal properties of the working fluids often used [5]. Nanofluids, dispersions of nano-sized solid particles in a base fluid, were conceived as thermally-improved heat transfer fluids [6,7,8], focusing the attention on thermal conductivity enhancements. Mechanisms such as agglomeration, liquid layering at the particle-fluid interface, fluid convection at the microscales, the thermophoretic effect, or in a lesser extent the Brownian motion have been reported as the cause of these enhancements, with contradictory conclusions [9]. Furthermore, during the last decade, the focus has also been put on the improvement of other properties for specific purposes in medical, solar, energy storage, electric, or magnetic applications [10,11,12]. Among these more recent research fields, their consideration as alternative working fluids for direct absorption solar thermal collectors has received increasing attention by the scientific community because of the great modifications of the original optical profiles, and higher sunlight absorption and extinction coefficients have been reached, even when using low nanoadditive concentrations [12,13,14,15,16].

From an economic point of view, it is relevant to control the required pumping power to make the fluid flow. This is basically influenced by the pressure drop through the installation, so is strongly dependent on the viscous transport characteristics of the fluid [17]. In heat transfer applications, the working fluid should present high thermal conductivity and low viscosity values [18,19]. Furthermore, for any of the previously described applications, study of the new performance obtained by nanofluids requires information on the flow regime conditions and the pumping power consumption. Slight improvements in the physical properties of the working fluid by the dispersion of nanoadditives, can have a huge impact on the required heat flux, the application design, and the budget [18]. Thus, viscosity and rheological characteristics play a critical role in any potential nanofluid application. Therefore, complete and rigorous studies substantiated on the rheological behavior become essential [20].

Carbon-based nanoadditives are available in many different types and shapes, and lots of them have been employed for nanofluids preparation. One of the most often used are nanotubes and the dynamic viscosity of nanofluids constituted by these types of materials are reported in numerous works [21,22,23,24,25,26]. Recently, other structures, like graphene nanoplatelets (GnP) and nanodiamonds, have become more widespread [27,28,29,30,31,32]. In our previous work [33], a comprehensive study on the dynamic viscosity of propylene glycol-water-based nanofluids containing sulfonic acid-functionalized GnP was presented. Newtonian behavior at higher shear rates with dynamic viscosity dependences on nanoadditive fraction and temperature was reported, as well as pseudoplasticity at lower shear rates. The rheological behavior of different-loaded polycarboxylate chemically modified GnP dispersions in water and propylene glycol:water mixtures was analyzed in [34], reporting viscosity values for Newtonian regimes with strong dependences on temperature and concentration. A linear viscoelastic region was also observed at the lowest deformations. In these previous works [33,34], a deep literature revision about rheological properties of water and glycolated water-based nanofluids containing graphene nanoparticles was carried out.

Minakov et al. [35] showed the shear thinning characteristic of water-based nanofluids containing nanodiamonds. They reported that viscosity increases with the increasing fraction, while non-Newtonian behavior was detected in the case of any examined concentration. A similar trend was observed by Żyła et al. [36] in ethylene glycol-based nanofluids containing nanodiamonds. They presented the results of a comprehensive study on the rheological behavior of nanofluids containing two types of nanodiamond particles with various purities. Beside the viscosity measurements, the viscoelastic structure and thixotropic behavior of those materials has also been investigated. Żyła et al. [37] have developed a rheological study about nanofluids containing a mixture of nanodiamond and graphite with various ash fractions. Non-Newtonian behavior and viscoelastic structure were also observed.

Another type of carbon nanoparticle used to prepare nanofluids in the literature are nanohorns. The dynamic viscosity of nanofluids containing a single wall carbon nanohorn was investigated by Selvam et al. [38]. They showed that suspensions containing a low volume fraction of particles could be considered as Newtonian fluids, while this behavior changes when the fraction increases.

In this work, the rheological profiles of twenty four ethylene glycol:water 50:50 vol%-based nanofluids designed using six different carbon-based nanoadditives were experimentally determined. A rotational rheometer coupled with a double cone geometry with a special cover to elude evaporation, sweeping different concentrations (0.25, 0.50, 1.0, and 2.0 wt%) and several temperatures (283.15 to 353.15 K, with 10 K steps), was employed for this purpose. The analysis of the flow curves in the 1–1000 s^−1^ shear rate range and the storage moduli, loss moduli, and complex viscosities as a function of the deformation in the 0.1–1000% range will allow the influences of different parameters of this type of nanofluid on their rheological characteristics to be described.

## 2. Experimental Section

### 2.1. Materials

The nanoadditives employed were six different carbon-based nanomaterials commercially named NanoDiamnods Purified grade G01 (PlasmaChem GmbH, Berlin, Germany), nD 97; NanoDiamnods Purified grade G (PlasmaChem GmbH, Berlin, Germany), nD 87; Graphite/Diamond Nano-Mixture Purified (PlasmaChem GmbH, Berlin, Germany), G/D p; Graphite/Diamond Nano-Mixture Raw (PlasmaChem GmbH, Berlin, Germany), G/D r; Carbon Black (PlasmaChem GmbH, Berlin, Germany), CB; and sulfonic acid-functionalized graphene nanoplatelets (NanoInnova Technologies S.L., Madrid, Spain), fGnP. The characteristics of each nanopowder, according to the manufacturer, are summarized in Table 1. Regarding the base fluid, ethylene glycol or ethane-1,2-diol, CAS number 107-211-1, was purchased from Honeywell (Seelze, Germany), with 99.5% declared purity, and was mixed with Milli-Q Grade water.

All the analyzed nanofluid samples were designed through a two-step method. The corresponding amounts of ethylene glycol and water required to obtain the desired volume fraction of 50:50 vol% and the necessary amounts of nanopowder required to reach the projected nanoadditive mass concentrations of 0.25, 0.50, 1.0, and 2.0 wt%, were weighted in an analytical balance WAS 220/X (Radwag, Radom, Poland), with an accuracy of 0.1 mg. With the aim of well-dispersing the nanoadditive into the base fluid, the mixtures were exposed to 200 min of ultrasonication with 450 W power and 45 kHz frequency by an ultrasound bath Emmi 60 HC (EMAG, Moerfelden-Walldorf, Germany).

### 2.2. Nanopowder Characterization

In order to characterize the size and the morphology of the six carbon-based nanoadditives employed, transmission electron microscopy (TEM) analyses were developed over drops of each nanopowder dispersion in analytical grade ethanol, previously dried under ambient conditions above the copper measuring support. A field emission transmission electron microscope JEM-2010F (JEOL, Tokyo, Japan) was employed, working at an operating accelerator voltage of 200 kW.

**Figure 1:**
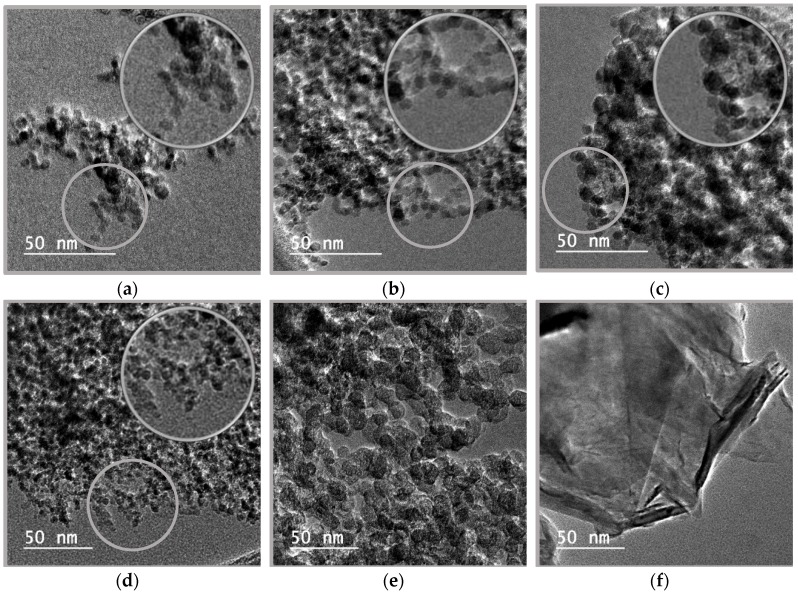
TEM images of the six carbon-based dry nanopowders employed: Nd 97 (**a**), Nd 87 (**b**), G/D p (**c**), G/D r (**d**), CB (**e**), and fGnP (**f**). The insets show selected areas enlarged by 50%.

Figure 1 shows the images obtained for each carbon-based nanopowder employed. It should be noted that all images use a different-scale reference line meaning 50 nm. Figure 1a,b correspond to diamond, one of the most stable carbon allotropes presenting a crystalline structure with carbon atoms tetrahedrally arranged and well-known for its great physical characteristics such as its high hardness and thermal conductivity. The insets show the quasi spherical nanoparticles with dimensions similar to the 4 nm declared by the manufacturer, with no evidence of different phase contents. Figure 1c,d show mixtures between the same nanodiamonds of Figure 1a,b, more clearly visible in the insets, and larger nanoparticles of graphite, the crystalline carbon allotrope known for its layered structure and for being the most present in nature. Figure 1e corresponds to carbon black, a form of amorphous carbon known for an extremely high surface-to-volume ratio. It shows nanoparticles with irregular forms and a dimension similar to that given by the manufacturer, 13 nm. Finally, Figure 1f corresponds to graphene, a carbon allotrope with the carbon atoms arranged in a hexagonal lattice in single layers and outstanding physical properties, like high hardness, flexibility, thermal and electrical conductivities. The figure evidences the nanoplatelet structure of stacked graphene sheets, with straight edges and dimensions in accordance with those previously described for this same commercial nanopowder in the literature [33,39], up to 500 nm.

### 2.3. Methods

The experimental tests designed to describe and analyze the rheological profiles of the selected carbon-based nanofluids were developed by a HAAKE MARS 2 rheometer (Thermo Electron Corporation, Karlsruhe, Germany), coupled with a double cone DC60/1 geometry (Thermo Electron Corporation, Karlsruhe, Germany) and a Peltier system matched with a HAAKE C25P refrigerated bath with a Phoenix II controller (Thermo Electron Corporation, Karlsruhe, Germany) that ensured the establishment of the set temperature with a 0.1 K accuracy. The selected geometry was composed of the double cone itself, which had a 60 mm diameter and 1° cone angle; the measuring base plate with a cylindrical wall in which the sample was located; and a stainless steel cover appropriate for avoiding evaporation at high measuring temperatures. The expanded uncertainty (*k* = 2) of the dynamic viscosity data by using this device was declared to be lower than 5.0% [36,40,41].

Rotational tests, allowing the non-linear behavior through viscosity versus shear rate curves once having reached the steady state to be described, were performed for the base fluid and all the designed nanofluids in the range of shear rates between 1 and 1000 s^−1^, with ten points per decade, and with temperature ranging from 283.15 to 353.15 K, with 10 K steps. Furthermore, oscillatory tests, permitting the linear viscoelastic behaviors to be analyzed by means of curves showing storage modulus, loss modulus, and complex viscosity as a function of deformation, were performed for the highest nanoadditive concentration, 2 wt%, of all the nanofluid sets in the range of deformations between 0.1 and 1000% and at 1 Hz and 293.15 K.

## 3. Results and Discussion

### 3.1. Experimental Validation

Firstly, in order to check the employed methodology, the comparison between the experimental dynamic viscosities obtained for the employed base fluid, ethylene glycol, and water mixture at a 50:50 vol% fraction (EG:W 50:50), and the literature data [42,43,44,45,46,47] has been carefully analyzed. The experimental dynamic viscosity values were considered as the average of the ten points of the 100 to 1000 s^−1^ shear rate decade of the corresponding flow curves. An evaluation of the apparatus working with the same double cone geometry was performed in a previous work [34] by determining the viscosities for water and propylene glycol:water mixtures, obtaining an absolute average deviation (AAD) of 2.5%.

**Figure 2:**
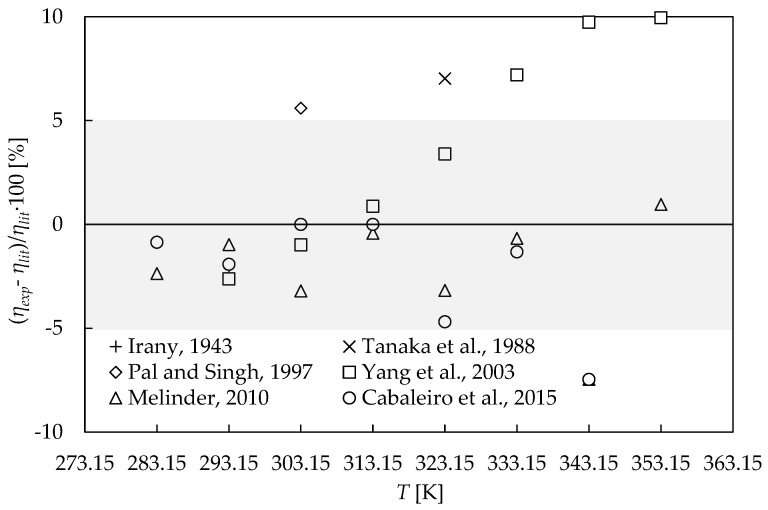
Percentage relative deviations among the experimental dynamic viscosities (*η*_exp_) and literature values (*η*_lit_) [42,43,44,45,46,47] for the ethylene glycol:water mixture 50:50 vol%. The grey area represents the declared expanded uncertainty (*k* = 2) region of the rheometer, 5.0%.

Figure 2 shows the percentage relative deviations between experimental and literature data [42,43,44,45,46,47] for the base fluid used in this work. The obtained AAD [42,43,44,45,46,47] is 3.4%, i.e., lower than the accuracy of the employed device. It should be noted that there are scattered values at high temperatures, with deviations between literature data of up to 16% at 343.15 K. However, overall deviations with those authors who present viscosities over a wide range of temperatures, including Yang et al. [45], Melinder [46], and Cabaleiro et al. [47], reach values of 4.9%, 2.4%, and 2.3%, respectively.

### 3.2. Rotational Rheology

**Figure 3:**
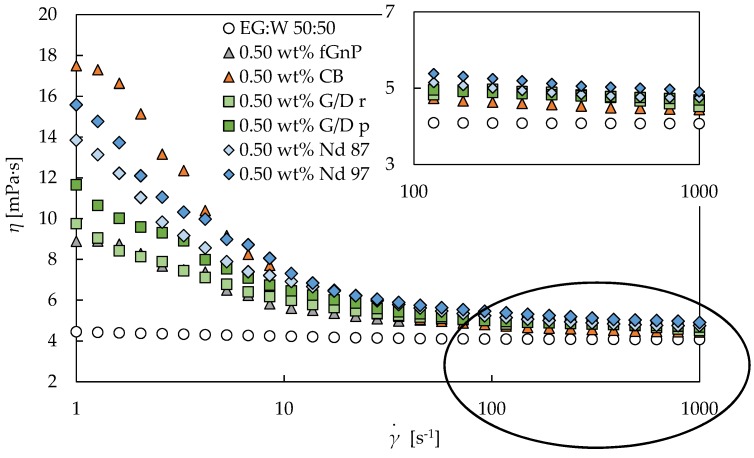
Experimental dynamic viscosities (*η*) obtained in the shear rate ($\dot{\gamma }$) range from 1 to 1000 s^−1^ for all types of 0.50 wt% nanofluids designed and the base fluid at 293.15 K. The inset shows the selected area amplified.

In this section, scrutiny of the rotational tests and non-linear behavior of the designed nanofluids are presented. Thus, the obtained experimental flow curves at different concentrations and temperatures are analyzed. Figure 3 shows the obtained data for the base fluid and six different nanofluid sets for the 0.50 wt% nanoadditives concentration at 293.15 K, as an example.

Figure 3 presents the non-Newtonian shear thinning or pseudoplastic behavior observed for all nanofluids at low shear rates in contrast to the well-known Newtonian behavior of the base fluid, EG:W 50:50 vol%. It can be observed that the first Newtonian plateau at the lowest shear rates is not appreciated for the different-phase Nd and the different-pure G/d nanofluids, while an incipient Newtonian region can be observed for the CB and fGnP nanofluids. The same behavior was detected for all the analyzed concentrations and temperatures. The appearance of shear thinning behavior for nanoadditive dispersions in Newtonian base fluids has been explained in the literature by the modifications in the arrangement of the involved agglomerates or particles [48,49,50]. According to this, some agglomerates of nanoadditives could break once oriented in the flow direction of the shear, reducing the quantity of bound solvent among the nanoparticles. Thus, the interaction forces could then be weaker, decreasing the flow resistance and, consequently, the apparent viscosity of the dispersion [48,49,50].

**Figure 4:**
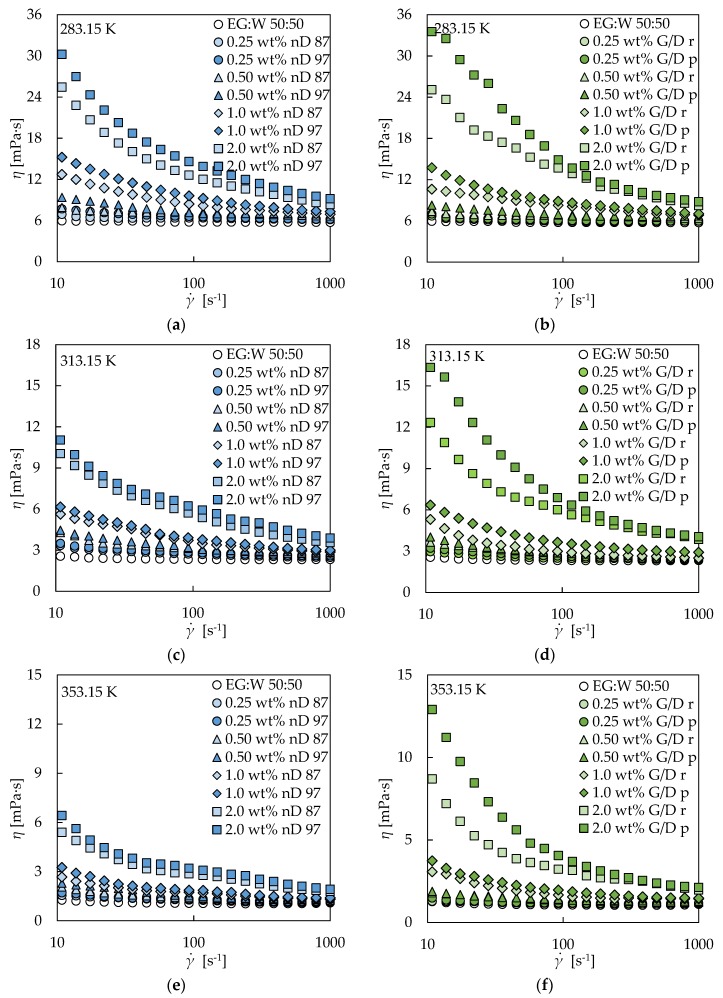
Experimental dynamic viscosities (*η*) obtained in the shear rate ($\dot{\gamma }$) range from 10 to 1000 s^−1^ for nD 87 and nD 97 nanofluids (**a,c**,**e**) and G/D r and G/D p nanofluids (**b,d**,**f**) at 283.15 K (**a,b**), 313.15 K (**c,d**), and 353.15 K (**e,f**).

**Figure 5:**
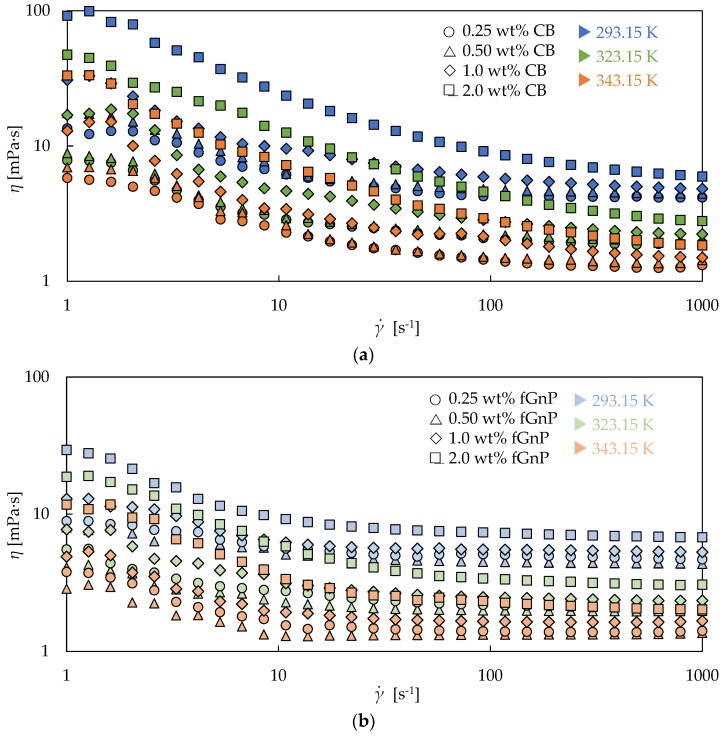
Experimental dynamic viscosities (*η*) obtained in the shear rate ($\dot{\gamma }$) range from 10 to 1000 s^−1^ for CB (**a**) and fGnP (**b**) nanofluids at 293.15 K, 323.15 K, and 343.15 K.

Figure 4 shows that the two different-phase nD nanofluid sets (Figure 4a,c,e) and the two different-pure G/d nanofluid sets (Figure 4b,d,f) exhibit a higher pseudoplasticity as the nanoadditive loading increases. As evidenced, this behavior was obtained at all the analyzed temperatures. The first Newtonian plateau is not observable for any concentration of the four nanofluid sets, as it was previously stated from Figure 3, while the second is only clearly evidenced for the two lowest, 0.25 and 0.50 wt%. On the other hand, Figure 5 shows that CB and fGnP sets also present a higher pseudoplasticity as the nanoadditive concentration rises for different analyzed temperatures. This behavior is consistent with the previous described theory for the shear thinning behavior of dispersions, with the agglomerates being more difficult to orient in the flow direction as the nanoadditive amount increases.

Figure 5a shows an incipient region of the first Newtonian plateau for all the concentrations of CB nanofluids at several temperatures. The second plateau is again only reached for the lowest concentrations, 0.25 wt% and 0.50 wt%, like for the Nd and G/D nanofluid sets. The figure shows that the dynamic viscosity decreases between both Newtonian plateaus in the range 220–370% for the 0.25 wt% nanofluid and in the range 280–420% for the 0.50 wt% nanofluid. It should be noted that the CB set shows the highest slope in the shear thinning region for all nanoadditive loadings, as Figure 3 depicts for the 0.50 wt% concentration. Figure 5b exhibits an emerging region of the first Newtonian plateau for all the concentrations, as the CB set, and also reaches the second Newtonian plateau for all the analyzed concentrations and temperatures, in contrast with the rest of the sets. The figure shows dynamic viscosity decreases between Newtonian plateaus in the ranges 82–120%, 83–170%, 140–220%, and 150–240% for the 0.25 wt%, 0.50 wt%, 1.0 wt%, and 2.0 wt% fGnP nanofluids, respectively. As observed, the highest decrease for fGnP nanofluids, at 2.0 wt%, is similar to the lowest decrease for CB nanofluids, at 0.25 wt%, which allows different magnitudes between the shear thinning behaviors to be observed.

Taking into account the different pureness samples, it can be noticed that generally, the discrepancies in the shear-thinning region are higher for the G/D nanofluids than for the Nd nanofluids. From Figure 4a,c,e, it can be pointed out that nanofluids constituted by the higher Nd phase present higher viscosity values than the ones construed by the lower Nd phase, with maximum differences of 13%, 18%, 29%, and 43% for the 0.25, 0.50, 1.0, and 2.0 wt% nanofluids, respectively. These results are in accordance with those presented by Zyła at al. [36] for ethylene glycol-based nanofluids containing the same two types of Nd. They showed the flow curves for various mass concentrations in the 1.0 wt% to 10 wt% range at 298.15 K, with slightly higher viscosity values for the higher Nd phase samples. Figure 3 and Figure 4b,d,f also show that in the case of the different-pure G/D nanofluids, the higher the purity, the higher the viscosity values of the corresponding nanofluids for the same concentration, with maximum differences of 17%, 29%, 36%, and 47% for the 0.25, 0.50, 1.0, and 2.0 wt% nanofluids, respectively. Zyła at al. [37] presented flow curves at 298.15 K for different mass concentrations between 1.0% and 5.0% of the same G/D nanopowders in ethylene glycol, with similar viscosities for the different pure samples. Furthermore, we can observe that for the highest concentrations and at lower shear rates, G/D nanofluids present higher dynamic viscosities than Nd nanofluids, while for the lowest concentrations, dynamic viscosities of Nd nanofluids are always higher than those of G/D nanofluids. The combined effect of graphite and nanodiamonds leads to lower viscosities than nanodiamonds at 0.25 wt% and 0.50 wt% concentrations, while for the highest, this trend starts to change.

Viscosity decreasing with increasing temperature was observed for all samples, as expected. The movement of the fluid particles is higher as the temperature increases, entailing a weakening of the inter-molecular cohesive forces and the consequent lower resistance of the fluid to flow [33,51]. It should be noticed that a quasi-constant relative decreasing was observed for the different carbon-based nanofluids, not dependent on concentration or shear rate. As an example, the dynamic viscosity values of the twenty-four nanofluids present decreases with the temperature ranging between 76 and 84% at a shear rate of 57.4 s^−1^, while at a shear rate of 489 s^−1^, the corresponding decreases range between 79 and 83%. It should be noted that these comparisons are made in some cases between the shear thinning region and Newtonian plateau, reinforcing the strength of this conclusion. This ~80% dynamic viscosity decrease for the 70 K step is similar to that of the base fluid, 82%. This behavior was also described in our previous work [34] for polycarboxylate chemically modified graphene nanoplatelet dispersions in water, a propylene glycol:water mixture at 30:70 wt%, and a propylene glycol:water mixture at 50:50 wt%, where nearly constant decreases for the same 70 K step of around 73%, 84%, and 88%, respectively, were described. These results would confirm the unique dependence of the temperature-related variations of viscosity on the base fluid for carbon-based nanofluids.

One of the most well-known equations to model Non-Newtonian behaviors in the flow curves is the Ostwald-de Waele model or Power law [52,53]:

(1) $$\eta =K\cdot {\dot{\gamma }}^{n-1}$$

where the fitting parameters *K* and *n* stand for the flow consistency index and the flow behavior index, respectively. The distance from 1 of *n* indicates the degree of deviation from Newtonianity. Thus, *n* < 1 denotes shear-thinning behavior and *n* > 1 denotes shear-thickening behavior [53]. Nevertheless, this equation strictly models the shear-thinning or shear-thickening regions, not including Newtonian plateaus. The entire shape of the flow curve for shear-thinning fluids can be defined by the Cross model [52,54]:

(2) $$\eta =\eta_{\infty }+\frac{\eta_{0}-\eta_{\infty }}{1+{\left(k\cdot \dot{\gamma }\right)}^{m}}$$

where *η*_0_ and *η_∞_* are the asymptotic values of viscosity corresponding to the Newtonian plateaus at lower and higher shear rates, respectively, while *k* and *m* are called the time constant and the rate constant, respectively [55,56]. The *m* value is related to the degree of dependence on the shear rate of the viscosity in the shear thinning region [57]. When *m* = 0, the system behaves as a Newtonian fluid [58]. On the other hand, the inverse of the time constant, 1/*k*, provides an order of magnitude of the critical shear rate for the end of the first Newtonian plateau and the onset of the shear-thinning region [57,59]. Other models that can be considered approximations of the Cross equation allow different parts of the curve to be described. The Sisko equation [60] is useful when *η*_0_ >> *η*, excluding the first Newtonian plateau of the modelling, and implies adding the *η_∞_* value in the right term of the Power law equation (*η* = *η_∞_* + *K*·$\dot{\gamma }$*^n^*^−1^). Nevertheless, the Williamson equation [61] is applicable when *η* > *η_∞_*, excluding the second Newtonian plateau of the modelling, and implies subtracting the right term of the Power law equation from the *η*_0_ value (*η* = *η*_0_ − *K*·$\dot{\gamma }$*^n^*^−1^).

The Power law equation, Equation (2), was employed to strictly model the shear thinning region in the 1 to 100 s^−1^ shear rate range of nD 97, nD 87, G/D p, and G/D r nanofluids at 298.15 K. As it can be observed in Table 2, low deviations were obtained for the lowest concentrations, 0.25 and 0.50 wt%, with AADs under 6.2% and 4.0% for the different-phase nD and the different-pure G/D nanofluids, respectively. Nevertheless, AAD increases as the concentration rises.

The *n* values obtained show a clear decreasing trend with the increasing concentration, evidencing a higher deviation from Newtonianity. Furthermore, the results are in accordance with the described behaviors with respect to the distinct pureness, with lower n values for the nanofluids constituted by the higher pure nanopowders. It is usually assumed that the flow behavior index varies between 0.2 and 0.8 for polymer melts and concentrated solutions [55]. It can be affirmed that our samples are within that range.

As the flow curves of the 0.25 and 0.50 wt% CB nanofluids and of all the different-concentrated fGnP nanofluids include the shear thinning region and both Newtonian plateaus, it was decided to test the accuracy of the Cross model, Equation (2), with the experimental data at 293.15 K. For each fitting process, the *η*_0_ value was directly assumed as the experimental dynamic viscosity at 1 s^−1^, while the *η_∞_* value was adopted as the average value of the dynamic viscosities in the 100–1000 s^−1^ range. AADs lower than 3.5% were reached for all nanofluids, as it can be observed in Table 3. It should be noted that the goodness of the adjustments gets worse as the nanoadditive concentration increases for both nanofluid sets.

**Figure 6:**
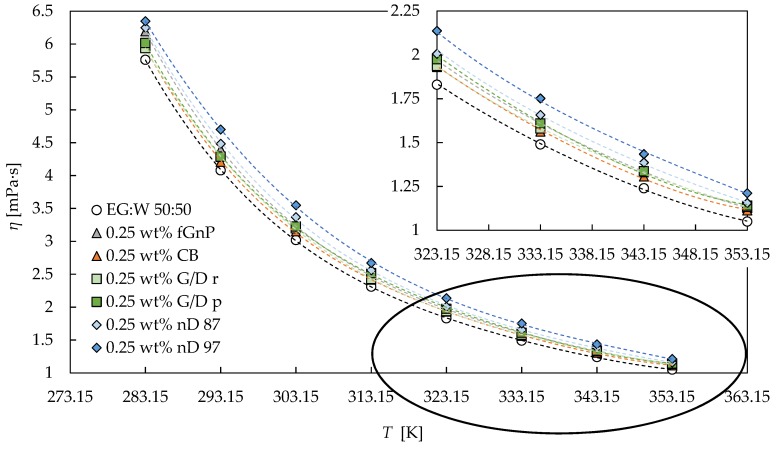
Dynamic viscosity (*η*) dependence on temperature (*T*) for all the 0.25 wt% nanofluids designed and the base fluid in the Newtonian 100–1000 s^−1^ shear rate range. The fitting lines correspond to VFT equation, Equation (3). The inset shows the selected area amplified.

The *m* values obtained show a higher displacement from zero with the increasing concentration, a symptom of more deviation from Newtonianity and a higher dependence on the shear rate of the viscosity values in the shear thinning region. Furthermore, the inverse of the time constant, 1/*k*, offers values between 4 and 5 s^−1^, in agreement with the critical shear rate between the first Newtonian plateau and shear-thinning region, as can be seen in Figure 5 and Figure 6 for the corresponding samples.

The higher shear rates analyzed in this study are the most connected with levels of turbulence of the usual flow rates in heat transfer applications. Thus, the effective dynamic viscosity values employed in the analysis of these processes correspond to the second Newtonian plateau for several of the analyzed nanofluids. As it was previously stated, it can be considered that for all the analysed carbon-based nanofluid sets, the 0.25 wt% and 0.50 wt% concentrations present quasi constant dynamic viscosity values in the 100–1000 s^−1^ shear rate range, while this can also be affirmed for the 1.0 and 2.0 wt% concentrations of fGnP. Thus, subsequently, the dynamic viscosity values of the second plateau were analyzed, considering them as the average of the experimental values in the cited shear rate range. Table 4 presents the aforementioned values for the 0.25 wt% and 0.50 wt% nanofluids. The following comparison between viscosity values can be established: *η_nD 97_* > *η_nD 87_* ≥ *η_G/D p_* > *η_G/D r_* > *η_CB_*, as can also be observed in Figure 6. The viscosity of the fGnP occupies different positions, depending on the concentration and temperature.

Average viscosity increases for the 0.50 wt% nanoadditive concentration with respect to the base fluid of 27%, 22%, 19%, 17%, 16%, and 13% for Nd 97, Nd 87, G/D p, G/D r, fGnP, and CB, respectively, were obtained. No clear temperature dependence of these increases was found. The dispersion of nanoparticles into a base fluid increases its internal resistance to flow due to the higher level of friction within it, leading to higher dynamic viscosity values [34,62].

The Vogel–Fulcher–Tammann (VFT) equation [63,64,65], also named the Vogel–Fulcher–Tammann–Hesse equation, is one of the most used models describing the temperature dependence of viscosity:

(3) $$\eta =\eta_{0}\cdot e^{\frac{A\cdot T_{0}}{T-T_{0}}}$$

where *η*_0_, *A*, and *T*_0_ are the fitting parameters. The results of the fitting process can also be observed in Table 4 for the base fluid and the 0.25 wt% and 0.50 wt% nanofluids. Low AADs between 0.17% and 1.3% were obtained, with standard deviations under 0.09 mPa·s.

**Figure 7:**
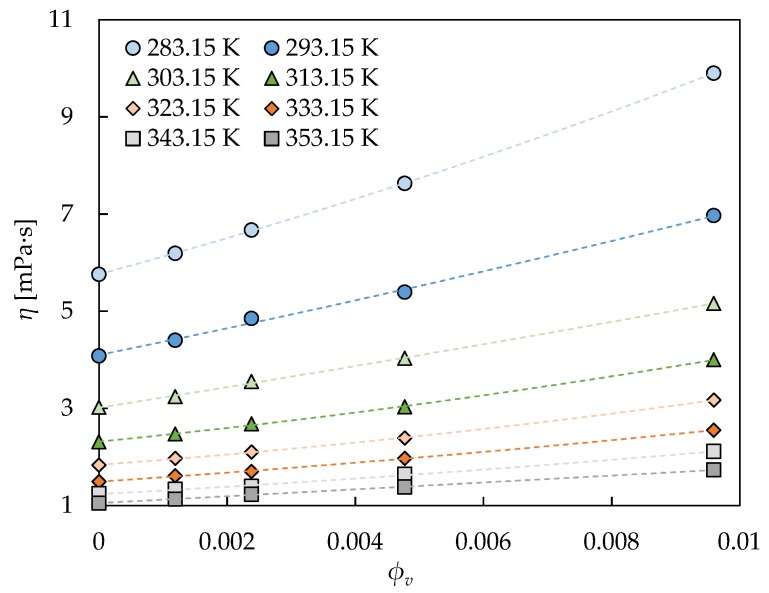
Dynamic viscosity (*η*) dependence on volume fraction (*ϕ_v_*) for the fGnP nanofluids in the second Newtonian plateau from 283.15 K to 253.15 K with 10 K steps. The fitting lines correspond to Vallejo et al.’s equation, Equation (4).

The dynamic viscosity values in the second plateau for the fGnP nafluid set were modelled by means of Vallejo et al.’s equation [33], which includes, in the same expression, the concentration and temperature dependences of viscosity:

(4) $$\eta =\eta_{0}\cdot e^{\frac{A\cdot T_{0}}{T-T_{0}}}+B\cdot e^{\frac{C}{T}}\cdot \;\phi_{v}\;-D\cdot \phi_{v}{}^{2}$$

with *B*, *C*, and *D* as the fitting parameters; *η*_0_, *A*, and *T*_0_ as the previously fitted parameters from the VFT equation, Equation (3), for the corresponding base fluid; and *ϕ_v_* as the volume fraction. This equation was tested in previous works [33,34,66] for experimental viscosities of GnP-water dispersions [34,39], GnP-propylene glycol water 10:90 wt% dispersions [33], GnP-propylene glycol water 30:70 wt% dispersions [33,34,66], and GnP-propylene glycol water 50:50 wt% dispersions [34], obtaining very good results. Table 5 and Figure 7 show the goodness of Equation (4) for the fGnP nanofluids of this study, the reached AAD being lower than 0.9%. The reduction in the number of employed fitting parameters with respect to the VFT equation for the entire nanofluid set (6 for Vallejo et al.’s equation versus 15 for VFT equations) should be noted. These results allow the validity of this equation to be tested for nanofluids when using an ethylene glycol:water mixture as a base fluid, and their good results for GnP as a nanoadditive to be consolidated.

The flow curves of fGnP nanofluids generally behave differently from the rest of the studied nanofluid sets, which are more similar among them despite the aforementioned differences. The fGnP nanofluid set presents the following particularities: the shear-thinning region is smaller than in other cases and the relative positions of the viscosity values at lower shear rates are different than at higher rates (see Figure 3 and Figure 6 and Table 4). The noticeable differences in the size and shape of fGnPs (up to 500 nm) are orders of magnitude larger than for the rest of the carbon-based nanomaterials (below 100 nm) employed, leading us to determine noticeable differences in the rheological behavior.

The small size of the training dataset leads to imprecise predictions of nanofluid thermophysical properties by artificial neural networks, making the conventional prediction models more suitable in many occasions [67,68]. The huge dataset provided by this study, 200 flow curves (25 different samples at eight temperatures), can be employed in future works to train neural networks for the prediction of nanofluids in different applications.

### 3.3. Oscillatory Rheology

**Figure 8:**
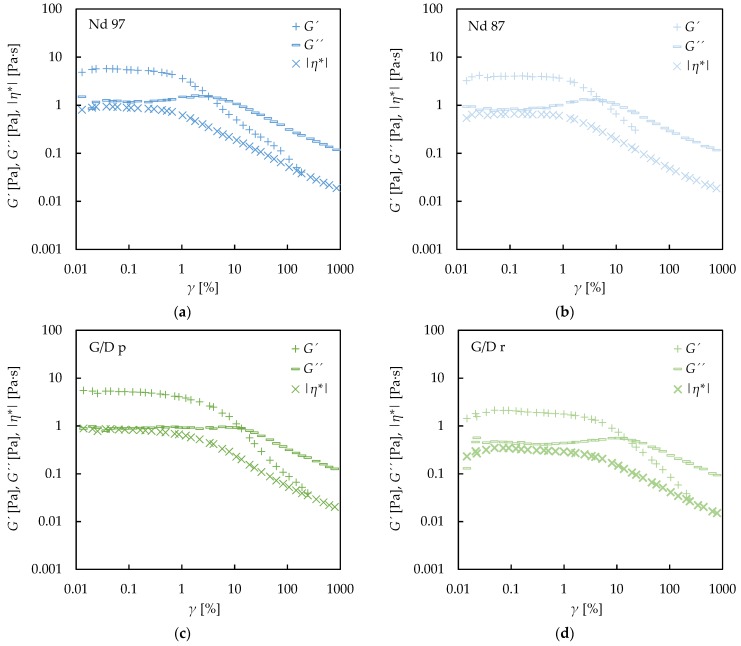

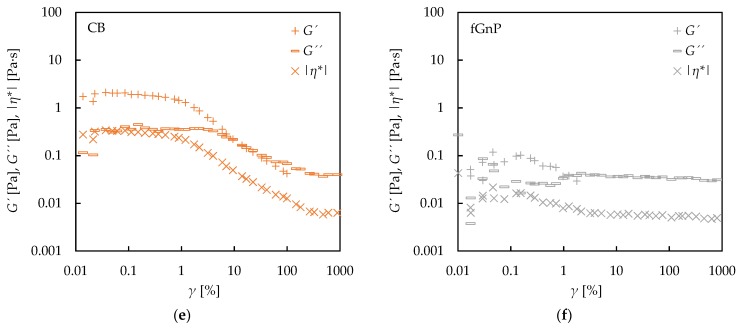
Storage modulus (*G′*), loss modulus (*G′′*), and complex viscosity (η*) dependence on percentage deformation (*γ*) for all the carbon-based 2.0 wt% nanofluids designed at 293.15 K: Nd 97 (**a**), Nd 87 (**b**), G/D p (**c**), G/D r (**d**), CB (**e**), and fGnP (**f**).

Figure 8 shows the results of an experimental oscillatory study on the various types of carbon-based nanofluids. The values of storage (*G′*) and loss (*G′′*) modulus as a function of deformation are presented at 1 Hz and 293.15 K, respectively. The complex viscosity, η* = (*G′* + i*G′′*)/*ω*, with *ω* being the angular frequency, is also shown. It might be noted that nanofluids containing fGnP exhibit the lowest value of complex viscosity, their viscoelastic structure is weak (low values of storage modulus), and no linear viscoelastic region can be described. In all other cases, viscoelastic behavior with a clearly visible range of the constant loss modulus value is observed. In nanofluids containing Nd, the value of storage modulus is similar, regardless of the purity of nanoadditives. In both nanofluids, the storage modulus starts decreasing at a value of deformation of approximately 1%. A more diverse behavior could be observed in the case of G/D nanofluids, for which the different pureness has a higher impact on the rheological behavior. The dispersions of purified particles exhibit higher values of storage modulus, loss modulus, and complex viscosity than the raw one. In those nanofluids, a linear viscoelastic region could also be observed, and the storage modulus starts decreasing at deformation higher than 1%. Nanofluids containing CB nanoparticles exhibit rheological behavior similar to that reported for Nd, but with lower values of storage, loss modulus, and complex viscosity.

The good agreement between the complex viscosity values at low deformations and 1 Hz frequency, and the results obtained in the flow curves at the lowest shear rate, 1 s^−1^, should be noted. Complex viscosities at the lowest deformations for the 2.0 wt% CB and fGnP nanofluids are the lowest, while these two nanofluid sets are the ones for which the first Newtonian plateau is observed.

## 4. Conclusions

The rheological behavior of different-loaded dispersions (0.25, 0.50, 1.0, and 2.0 wt%) of six carbon-based nanomaterials (carbon black, two different phase content nanodiamonds, two different purity graphite/diamond mixtures and sulfonic acid-functionalized graphene nanoplatelets) in ethylene glycol:water mixture 50:50 vol% have been experimentally analyzed. A deviation between experimental and literature viscosity data for the base fluid of 3.4% over the 283.15–353.15 K range was obtained. Shear thinning non-Newtonian behavior was found for all the carbon-based nanofluid sets. The higher pureness of the nanodiamond and graphite/diamond nanofluids leads to higher viscosity values, with the differences being higher in the second case. The comparison between viscosity values at high shear rates is the following: *η_nD 97_* > *η_nD 87_* ≥ *η_G/D p_* > *η_G/D r_* > *η_CB_*. The viscosity of the fGnP occupies different positions, depending on the concentration and temperature. The decreases of the dynamic viscosity are located around 80% in the analyzed temperature range for all carbon-based nanofluids, and 82% for the base fluid. The second Newtonian plateau is reached for all nanofluid sets at the two lowest concentrations, while both Newtonian plateaus were found for all fGnP nanofluids and the two lowest loadings of CB. Shear thinning regions were well-described using the Ostwald-de Waele model and Cross model, while Vallejo et al.’s equation allowed *η*(*T*,*ϕ_v_*) curves corresponding to the second Newtonian Plateau of the fGnP nanofluids with deviations of less than 0.9% to be described. Oscillatory tests evidenced the viscoelastic behavior for samples with the clearest Non-Newtonian response. The clear size and shape discrepancies between fGnP and the rest of the carbon-based nanomaterials employed decisively show differences in their rheological profiles.

## Figures and Tables

**Table 1:** Main characteristics of the employed nanoadditives, according to the manufacturers ^1,2^. NanoDiamnods Purified grade G01 (nD 97), NanoDiamnods Purified grade G (nD 87), Graphite/Diamond Nano-Mixture Purified (G/D p), Graphite/Diamond Nano-Mixture Raw (G/D r), Carbon Black (CB), and sulfonic acid-functionalized graphene nanoplatelets (fGnP).

Sample	Average Particle Size	Diamond Content	Non-Diamond Carbon Content	Ash Content	Specific Surface Area
nD 97 ^1^	4 nm	>97 wt%	traces ^3^	<1.4 wt%	350 m^2^·g^−1^
nD 87 ^1^	4 nm	>87 wt%	<6 wt% ^4^	<6 wt%	290 m^2^·g^−1^
G/D p ^1^	4 nm (diamond)	>20 wt%	-	<0.3 wt%	470 m^2^·g^−1^
G/D r ^1^	4 nm (diamond)	>20 wt%	-	<6 wt%	370 m^2^·g^−1^
CB ^1^	13 nm	-	-	<0.02 wt%	550 m^2^·g^−1^
fGnP ^2^	-	-	-	-	-

^1^ PlasmaChem GmbH, Berlin, Germany. ^2^ NanoInnova Technologies S.L., Madrid, Spain. ^3^ Fe < 0.3 wt%; Cu < 0.01 wt%; Zn < 0.01 wt%; Mn < 0.01 wt%; Si + Cr + Ca + Ti < 0.01 wt%.^4^ Fe < 1.2 wt%; Ca + Zn + Cr + Ni + Cu + Mn < 2 wt%.

**Table 2:** Fitting parameters (*K*, *n*), standard deviations (s), and absolute average deviations (AAD) obtained from the Power law equation, Equation (2), modelling the 1 to 100 s^−1^ shear rate range for the analysed mass fractions, *ϕ_m_*, of the nD 97, nD 87, G/D p, and G/D r nanofluid sets at 298.15 K.

Nanoadditive	*ϕ_m_*	*K* (mPa·s*^n^*)	*n*	s (mPa·s)	AAD
nD 97	0.0025	9.39	0.831	0.31	4.0%
	0.0050	14.2	0.755	0.67	5.9%
	0.010	22.3	0.695	1.4	8.4%
	0.020	83.3	0.491	10.6	18%
nD 87	0.0025	8.53	0.853	0.33	4.5%
	0.0050	11.9	0.798	0.80	6.1%
	0.010	19.5	0.721	0.67	4.9%
	0.020	54.9	0.564	8.2	15%
G/D p	0.0025	6.04	0.921	0.19	2.0%
	0.0050	11.0	0.802	0.33	3.9%
	0.010	24.8	0.654	1.4	8.0%
	0.020	108	0.437	6.4	14%
G/D r	0.0025	5.28	0.959	0.10	1.1%
	0.0050	9.06	0.848	0.30	3.6%
	0.010	20.2	0.686	0.75	6.4%
	0.020	68.3	0.538	4.1	8.3%

**Table 3:** Fitting parameters (*η*_0_, *η_∞_*, *k*, *m*), standard deviations (s), and absolute average deviations (AAD) obtained from the Cross model, Equation (1), modelling the 1 to 1000 s^−1^ shear rate range for the suitable mass fractions, *ϕ_m_*, of the CB and fGnP nanofluid sets at 298.15 K.

Set	*ϕ_m_*	*η*_0_ (mPa·s)	*η*_∞_ (mPa·s)	*k* (s)	*m*	s (mPa·s)	AAD
CB	0.0025	13.5	4.21	0.204	1.52	0.41	2.9%
	0.0050	17.5	4.56	0.250	1.58	0.57	3.5%
fGnP	0.0025	7.99	4.40	0.202	1.51	0.19	1.6%
	0.0050	8.89	4.85	0.212	1.77	0.16	2.2%
	0.010	12.9	5.39	0.250	1.82	0.29	2.4%
	0.020	25.2	6.97	0.259	1.91	0.46	3.0%

**Table 4:** Dynamic viscosity values (*η*) in the second Newtonian plateau for 0, 0.0025, and 0.0050 nanoadditive mass fraction, *ϕ_m_*, dispersions and fitting parameters (*η*_0_, *A*, and *T*_0_), standard deviation (s), and absolute average deviation (AAD) obtained from te VFT equation, Equation (3).

*ϕ_m_*	Set	Temperature (K)								Fitting Parameters and Deviations				
		283.15	293.15	303.15	313.15	323.15	333.15	343.15	353.15	*η*_0_ (mPa·s)	*A*	*T*_0_ (K)	*s* (mPa·s)	AAD
0	-	5.76	4.08	3.02	2.31	1.83	1.49	1.24	1.05	0.0413	4.37	150.25	0.006	0.17%
0.0025	nD 97	6.35	4.70	3.55	2.67	2.14	1.75	1.43	1.21	0.0323	6.03	132.61	0.082	0.85%
	nD 87	6.25	4.48	3.37	2.56	2.01	1.66	1.39	1.16	0.0322	5.79	134.87	0.022	0.64%
	G/D p	6.02	4.29	3.22	2.51	1.98	1.61	1.34	1.14	0.0395	4.97	142.42	0.014	0.27%
	G/D r	5.94	4.28	3.20	2.43	1.94	1.58	1.33	1.13	0.0363	5.29	138.98	0.023	0.65%
	CB	5.95	4.21	3.15	2.42	1.93	1.56	1.31	1.11	0.0371	5.06	141.69	0.024	0.43%
	fGnP	6.19	4.40	3.24	2.47	1.97	1.61	1.33	1.13	0.0438	4.39	150.15	0.014	0.34%
0.0050	nD 97	7.01	5.12	3.82	2.93	2.34	1.91	1.61	1.38	0.0350	6.38	128.42	0.030	0.87%
	nD 87	6.92	4.89	3.68	2.87	2.20	1.84	1.51	1.31	0.0355	5.90	133.49	0.046	1.1%
	G/D p	6.69	4.82	3.58	2.71	2.20	1.77	1.50	1.31	0.0409	5.25	139.48	0.040	1.1%
	G/D r	6.58	4.79	3.50	2.70	2.13	1.76	1.45	1.26	0.0375	5.42	138.14	0.040	0.91%
	CB	6.53	4.56	3.35	2.61	2.08	1.71	1.42	1.16	0.0394	5.08	141.68	0.072	1.1%
	fGnP	6.67	4.85	3.55	2.68	2.10	1.70	1.39	1.23	0.0191	8.09	118.89	0.051	1.3%

**Table 5:** Fitting parameters (*η*_0_, *A*, *T*_0_, *B*, *C*, and *D*), standard deviation (s), and absolute average deviation (AAD) obtained from Vallejo et al.’s equation, Equation (4), for the fGnP nanofluid set.

*η*_0_ (mPa·s)	*A*	*T*_0_ (K)	*B* (mPa·s)	*C* (K)	*D* (mPa·s)	s (mPa·s)	AAD
0.0413	4.37	150.25	0.0131	2916.07	2821.67	0.040	0.89%

## Abbreviations

**Nomenclature**	
AAD	Absolute average deviation
*ω*	Angular frequency
CB	Carbon Black
*η**	Complex viscosity
*γ*	Deformation
*η*	Dynamic viscosity
EG:W 50:50	Ethylene glycol:water 50:50 vol% mixture
*η*_0_, *η_∞_, k, m*	Fitting parameters from Cross model, Equation (2)
*K, n*	Fitting parameters from Ostwald-de Waele model, Equation (1)
*B, C, D*	Fitting parameters from Vallejo et al.’s model, Equation (4)
*η*_0_, *A, T*_0_	Fitting parameters from VFT model, Equation (3)
GnP	Graphene nanoplatelet
G/D	Graphite/diamond mixture
G/D p	Graphite/Diamond Nano-Mixture Purified
G/D r	Graphite/Diamond Nano-Mixture Raw
*G″*	Loss modulus
wt%	Nanoadditive mass concentration, %
*ϕ_m_*	Nanoadditive mass fraction
vol%	Nanoadditive volume concentration, %
*ϕ_v_*	Nanoadditive volume fraction
Nd	Nanodiamond
nD 97	NanoDiamnods Purified grade G01
nD 87	NanoDiamnods Purified grade G
$\dot{\gamma }$	Shear rate
s	Standard deviation
*G′*	Storage modulus
fGnP	Sulfonic acid-functionalized graphene nanoplatelets
*T*	Temperature
TEM	Transmission electron microscopy
VFT	Vogel-Fulcher-Tammann
**Subscripts**	
*exp*	Experimental value
*lit*	Literature value
